# HOW DOES TELEMEDICINE FIT INTO HEALTHCARE TODAY?

**DOI:** 10.1590/0102-672020210003e1584

**Published:** 2022-01-05

**Authors:** Gustavo ISOLAN, Osvaldo MALAFAIA

**Affiliations:** 1 Principles of Surgery Postgraduate Program at the Mackenzie Evangelical College of Paraná in Curitiba, Brazil

Telemedicine began at the Massachusetts General Hospital in Boston, USA, in the early 1970s to extend medical care to rural areas, and it initially focused on radiology. Technological innovation led to new options for healthcare, and the idea was born that doctors could discuss cases, review exams, and even examine patients without traveling from one hospital to another. Since then, telemedicine has drawn the interest of all types of healthcare organizations that want to extend their services to regions lacking in specialists. 

Remote consultation between physicians in Brazil had already been regulated by federal resolution since 2002, and in early 2020^4^, the Ministry of Health issued a decree that regulates remote medical care between doctors and patients during the Coronavirus pandemic^1^. It is expected that after this pandemic, the remote care of patients will continue to be authorized by the government. 

While there is an urgent need to provide specialized medical care to underserved patients in isolated regions, professional medical organizations warn of telemedicine´s potential risks and shortcomings. At the same time, the Federal Health Ministry urgently wants to reduce costs by eliminating the unnecessary transfer of patients between clinics. Market forces also play a role. Some organizations create artificial regulatory hurdles that oppose telemedicine, and sometimes quite unreasonably. Others call for complete deregulation that would lower medical costs but also allow for the growth of dubious services.

For example, neurosurgeons and orthopedists will certainly encounter technical barriers to accurately diagnosing a patient with spinal pain even if they have access to the most advanced exams on one computer screen while talking directly with the patient on another. To our understanding, a precise topographic diagnosis and etiology of such cases cannot be reliably achieved through telemedicine alone. This requires certain hands-on tests to be performed while interacting the patient. Testing a reflex, the sensitivity of a certain dermatome, or the strength of a particular muscle cannot be performed by patients themselves or by non-medical professionals even with the assistance of telemedicine technology. The same restriction applies to diagnosing peritoneal irritation, signs of meningitis, or signs of onset or fully acute abdomen, among many others. Most diagnostic techniques are still not supported with a high degree of clinical and epidemiological evidence. Although the medical literature is broad and growing exponentially, the presence of the physician is crucial to investigating a large number of diseases and symptoms. 

Even considering the above, it cannot be denied that telemedicine is a miracle in terms of saving lives and minimizing poor outcomes. To name a few examples: What to do with a patient suffering from acute ischemic stroke who is 200 kilometers away from the nearest neurologist and has a thrombolytic treatment window of less than an hour? Or how to reduce the many needless referrals that would be better handled at the hospital of origin, and which also burden the resources and budgets of emergency care and transportation systems? Or how to help a patient who reports atypical chronic headache and who lives three days by boat from the nearest specialist? For these neurological emergencies as well as for screening patients to assess the need for referral, telemedicine is perhaps the best solution. The cases in these examples, however, have already been authorized for remote consultations between doctors since 2002^1^^,^ 2 ^,^ 3 ^,^ 4 ^,^ 5 ^,^ 6 ^,^ 7 ^,^ 8 ^,^ 9 ^,^ 10. The crucial debate emerging now revolves around the need for scientific validation of what distance care could offer remote doctor-patient consultation, and also what inherent risks this holds for certain disorders. Without external validation and rigorous analysis, the arguments surrounding this will always be hobbled by ideology and preference. Sufficient pier-reviewed studies of telemedicine, when compared with in-person treatments, will resolve these debates. What is more, these should also evaluate the sensitivity, specificity, and positive and negative predictive values for each sign and symptom evaluated by telemedicine. 

Medicine is science and art. We express art through our individual creativity, from our empathy that calms our patients’ souls to our unique abilities to treat them on the operating table. Science, on the other hand, requires constant validation and is one of the foundations of good medicine. The time has come for telemedicine to be justly evaluated by the scientific method. 
